# Supplementary material for: Development of a High Throughput Screen for the Identification of Inhibitors of Peptidoglycan O-Acetyltransferases, New Potential Antibacterial Targets
Source: Antibiotics (Basel). 2019 May 27;8(2):65. doi: 10.3390/antibiotics8020065 (PMC6627197; doi:10.3390/antibiotics8020065)
Supplement: Supplementary file 1 [file antibiotics-08-00065-s001.pdf]

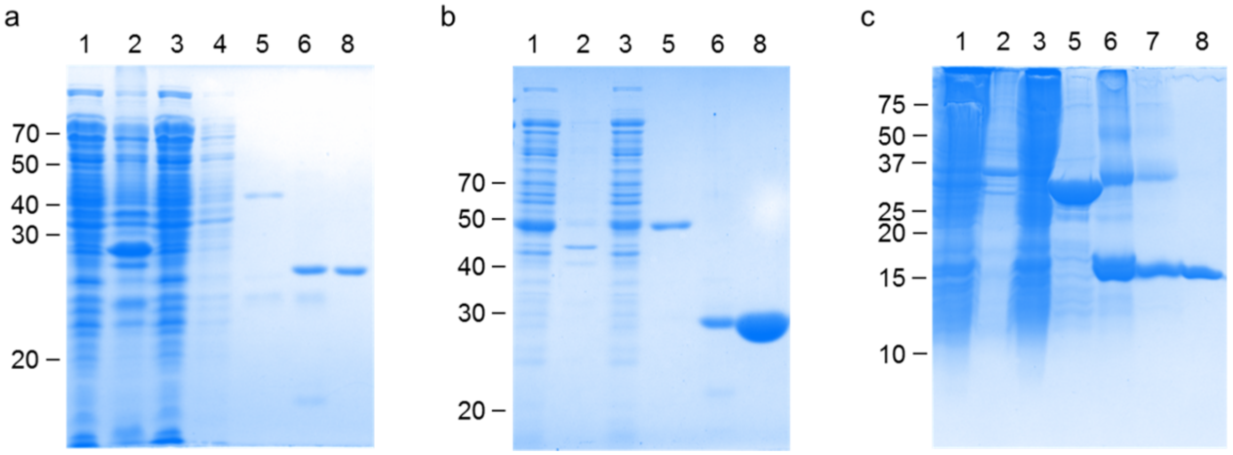

Figure S1. SDS PAGE analysis of the purification of (a) *Ng*PatB$_{\Delta 69}$, (b) *Ng*PatB$_{\Delta 100}$, and (c) *Sa*OatA$_{c\Delta 445}$. Lanes: 1, clarified soluble lysate; 2, insoluble lysate; 3, flow through after incubation with Ni^{2+} affinity resin; 4, wash fraction of Ni^{2+} affinity resin with 10 mM wash buffer; 5, Ni^{2+} affinity resin elution fraction; 6, elution following SUMO protease digestion; 7, SUMO digestion cleavage capture; and 8, final prepared sample purified by size exclusion chromatography (for *Sa*OatA$_{c\Delta 445}$), or cation-exchange chromatography (for *Ng*PatB$_{\Delta 69}$ and *Ng*PatB$_{\Delta 100}$). These purified proteins were stored for at least a month prior to the analysis presented to demonstrate their long-term stability. Note, not all fractions are present in each respective gel. Molecular weight markers (kDa) are indicated on the left.

Table S1. Kinetic analysis of *Sa*OatA and *Ng*PatB variants as *O*-acetylesterases. [1]

Substrate	Variant	K_M (mM)	k_{cat} (sec^{-1})	k_{cat}/K_M (M^{-1}·sec^{-1})
**4MU-Ac**	*Ng*PatB$_{\Delta 69}$	0.49 ± 0.1	0.06 ± 0.003	122 ± 26
	*Ng*PatB$_{\Delta 100}$	0.49 ± 0.06	0.048 ± 0.002	98.3 ± 13
	*Sa*OatA$_{\Delta 445}$	0.11 ± 0.01	0.0047 ± 0.00003	41.8 ± 4.4
***p*NP-Ac**	*Ng*PatB$_{\Delta 69}$	1.77 ± 0.19	0.14 ± 0.006	79.1 ± 9.1
	*Ng*PatB$_{\Delta 100}$	2.96 ± 0.07	0.44 ± 0.006	214 ± 8.0
	*Sa*OatA$_{\Delta 445}$	1.14 ± 0.05	0.047 ± 0.0008	41.2 ± 2.0

[1] The Michaelis-Menten steady-state kinetic parameters were determined for 3 µM *Ng*PatB in 50 mM sodium phosphate buffer, pH 7.0, and for 5 µM *Sa*OatA$_{\Delta 445}$ in 50 mM sodium phosphate buffer, pH 6.5 using 0.005–5 mM *p*NP-Ac, and 0.02–2 mM 4MU-Ac as substrates. ± standard deviation (n=3.

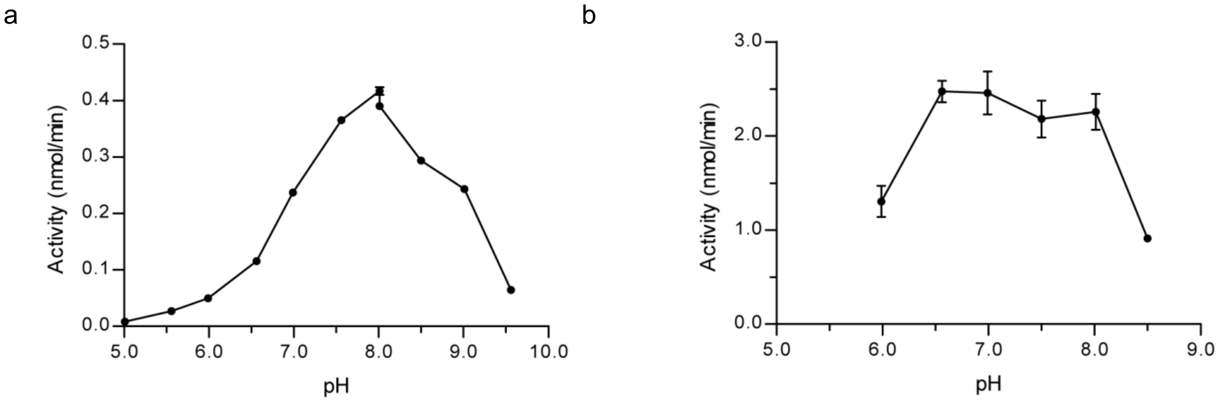

Figure S2. Dependence of (a) *Sa*OatA$_{\Delta 445}$ and (b) *Ng*PatB$_{\Delta 100}$ *O*-acetylesterase activity on pH. 4MU-Ac in 50 mM tripartite buffer consisting of sodium citrate, sodium phosphate and sodium borate was used as substrate to assay the *O*-acetylesterase activity of both enzymes at the pH values indicated.

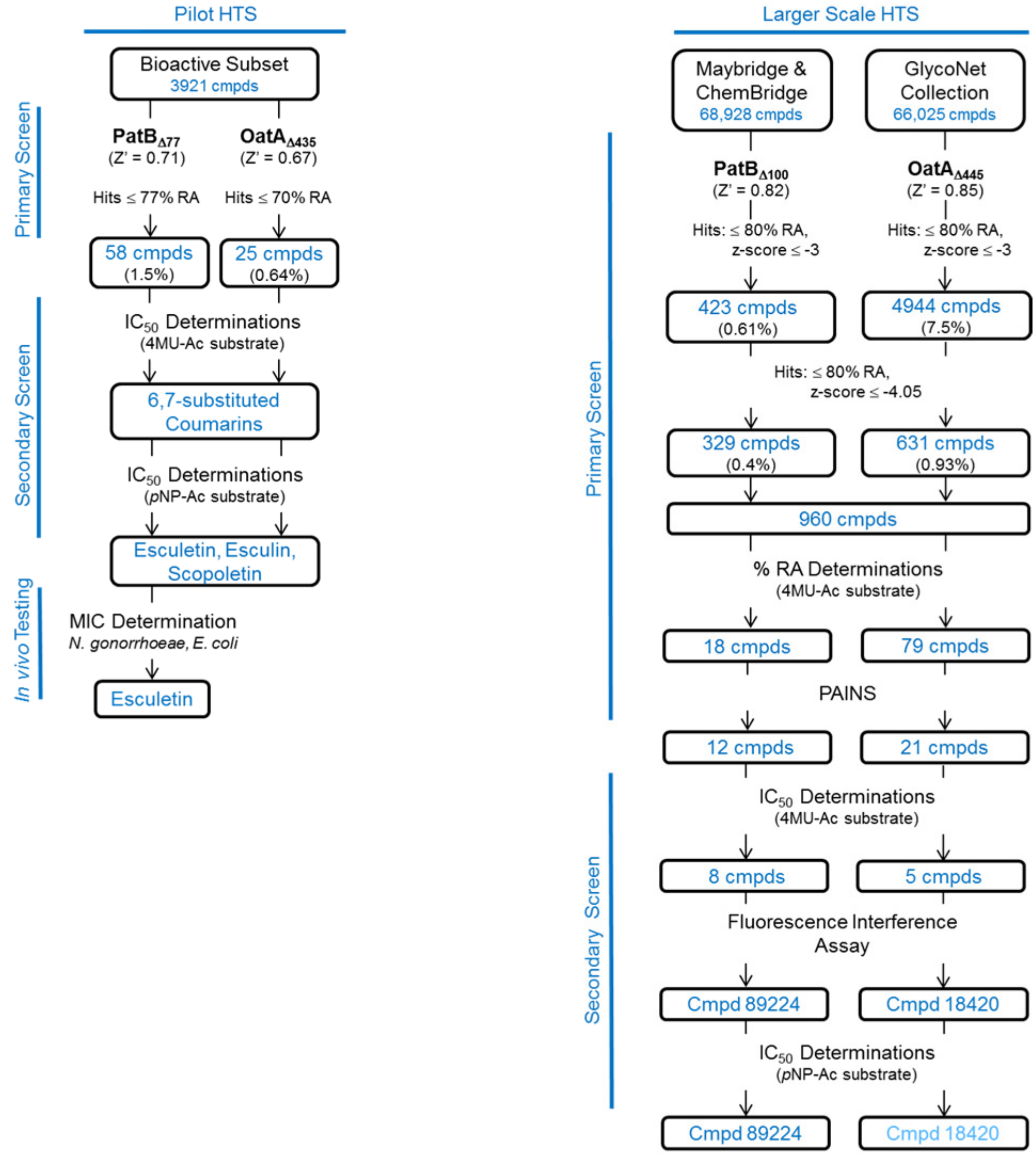

Figure S3. Flowcharts of pilot and larger-scale screens for inhibitors of PatA and OatA.

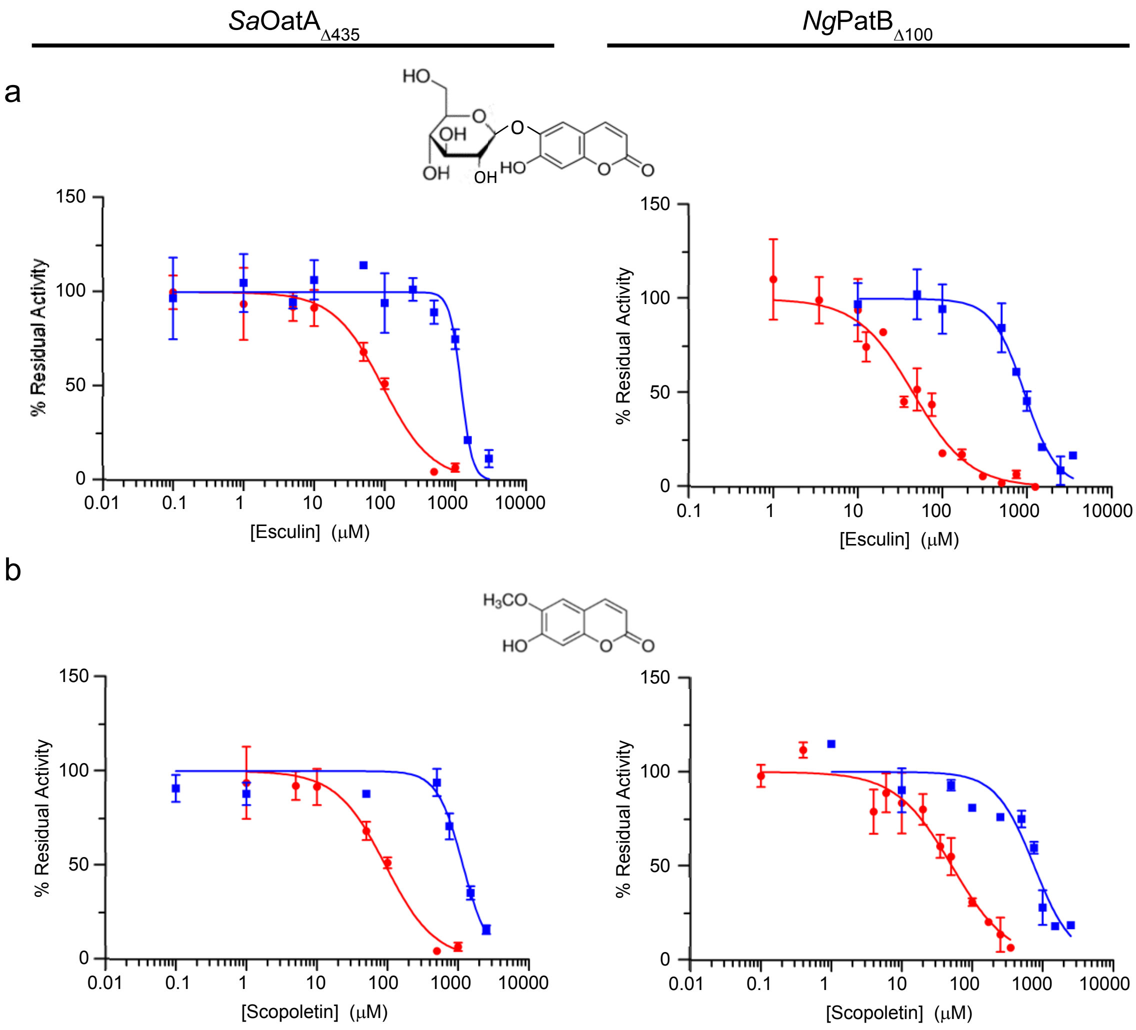

Figure S4. Molecular structure and half maximal inhibitory concentrations of (a) esculin and (b) scopoletin for inhibition of *Sa*OatA$_{\Delta 435}$ and *Ng*PatB$_{\Delta 69}$. The dose response assays were conducted on both enzymes using 4MU-Ac (red) and *p*NP-Ac (blue) as substrates. Error bars denote standard deviations, (n = 3).

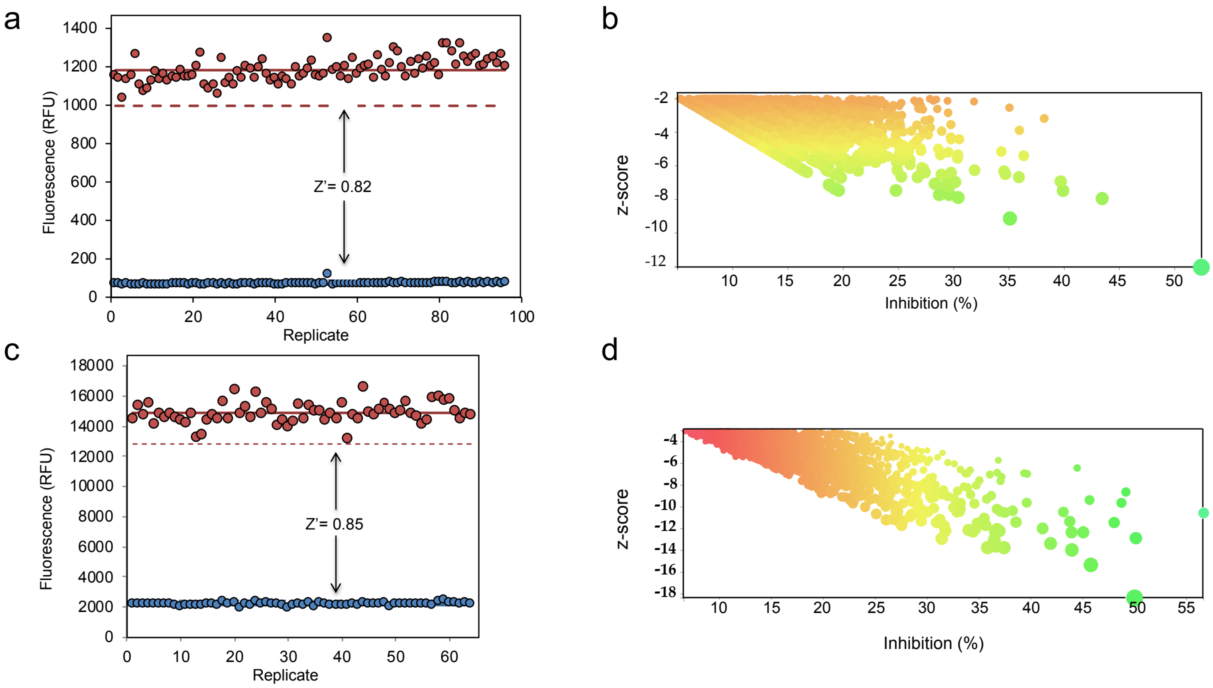

Figure S5. Large scale HTS for inhibitors of *Sa*OatA$_{\Delta 445}$ and *Ng*PatB$_{\Delta 100}$. Determination of *Z′* values for the large scale HTS assays of (a) *Sa*OatA$_{\Delta 445}$ and (c) *Ng*PatB$_{\Delta 100}$ esterase activity. Results of the primary screens of (b) *Sa*OatA$_{\Delta 445}$ and (d) *Ng*PatB$_{\Delta 100}$ tested against separate libraries of over 60,000 compounds each. The heat maps serve to identify hits (green) which have both low z-scores and strong inhibition compared to in-plate controls.

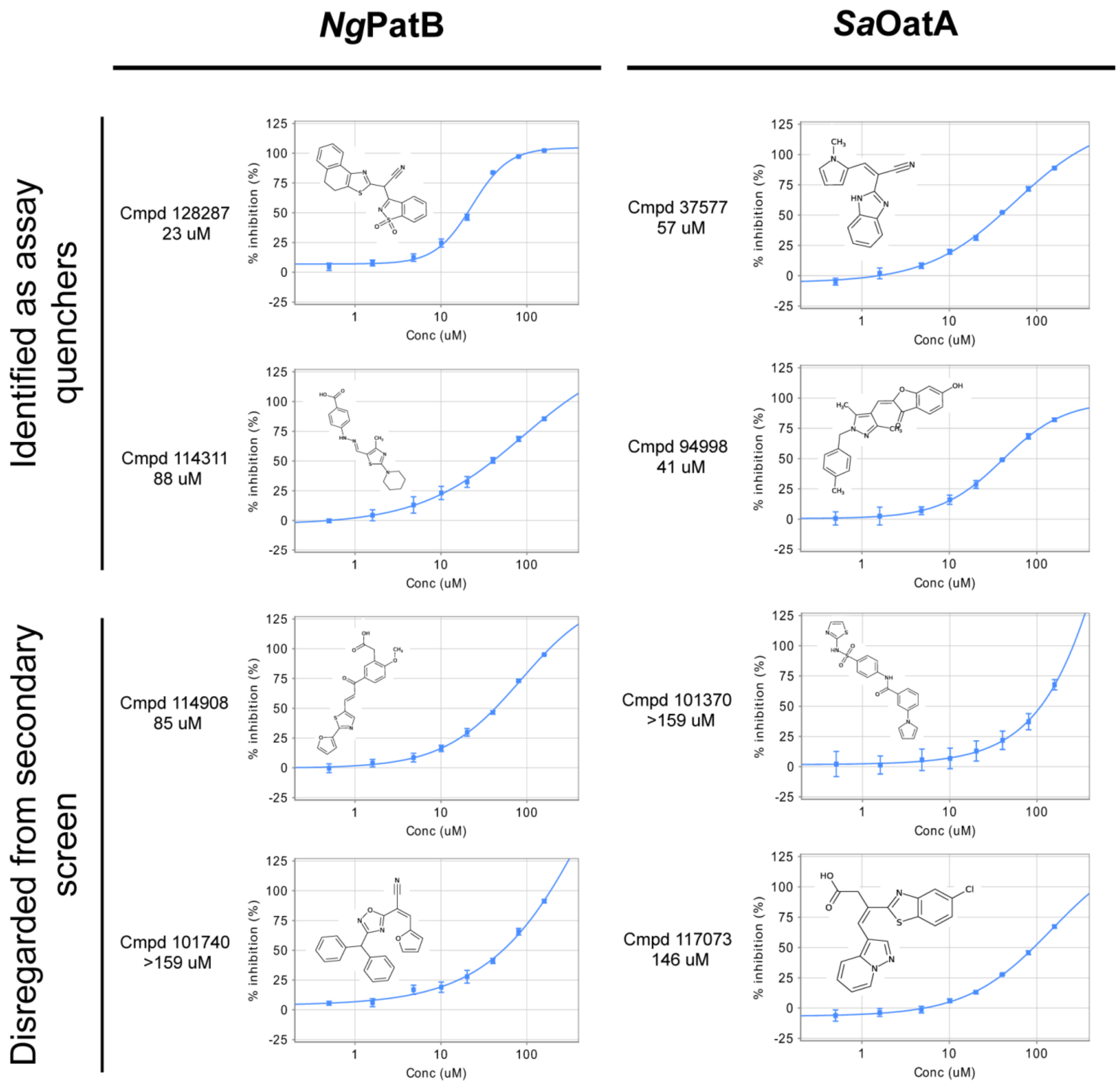

Figure S6. Representative dose response curves of *Ng*PatB$_{\Delta100}$ and *Sa*OatA$_{\Delta445}$ inhibition in the presence of select potential hit compounds from the respective large-scale screens that help to identify false-positives. 4MU-Ac was used as the substrate for these IC_{50} determinations. The error bars denote standard deviation (n=3).

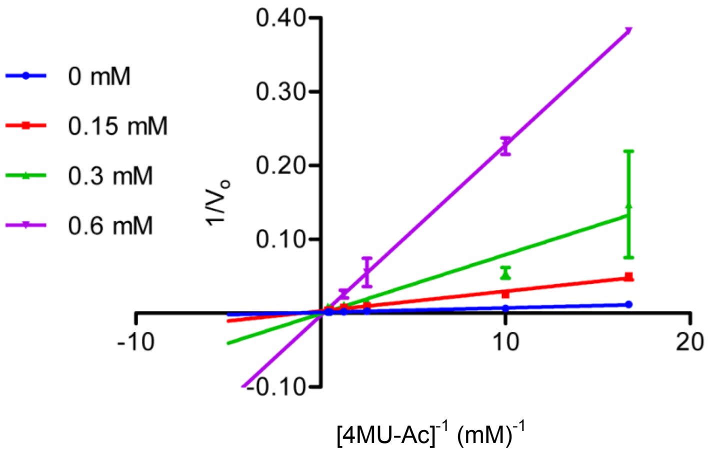

Figure S7. Mode of inhibition of *Ng*PatB$_{\Delta 69}$ by esculetin. Double reciprocal plot of initial velocity *vs*. substrate concentration in the presence of esculetin at the concentrations indicated. Error bars denote standard deviation (n=3).

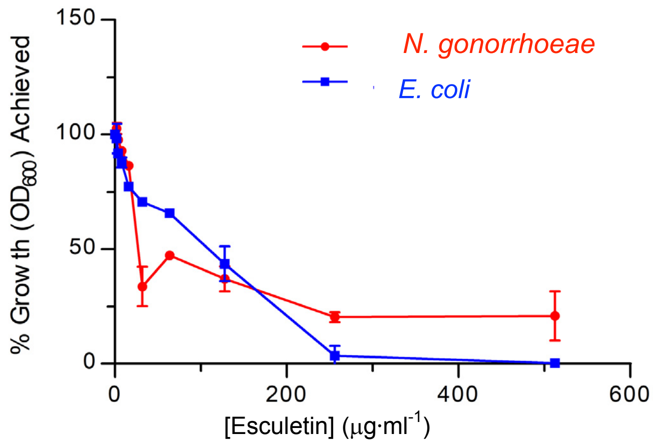

Figure S8. Effect of esculetin on bacterial cell growth. Cultures of *N. gonorrhoeae* and *E. coli* in their respective liquid growth media were incubated at 37 °C with esculetin at the concentrations indicated and cell growth was monitored at 600 nm until control cultures reached stationary phase. Growth (%) was normalized to solvent controls (0 µg·ml^{-1} esculetin); solvent concentration was held constant at 5% (v/v). Error bars denote standard deviations (n = 3).
